# SBN60, strontium-barium niobate at 100 K

**DOI:** 10.1107/S1600536813025142

**Published:** 2013-09-28

**Authors:** Marcin Stachowicz, Olga Gawryszewska, Marek A. Swirkowicz, Tadeusz Lukasiewicz

**Affiliations:** a Chemistry Department, Uniwersity of Warsaw, 1 Pasteura Str., 02-093, Warsaw, Poland; b Institute of Electronic Materials Technology, 133 Wolczynska Str., 01-919 Warsaw, Poland

## Abstract

The title compound, Sr_0.6_Ba_0.4_Nb_2_O_6_ (strontium barium niobium oxide), belongs to the group of strontium–barium niobates with varying composition of Sr and Ba. Their general formula can be written as Sr_*x*_Ba_1 - *x*_Nb_2_O_6_. Below the Curie temperature, *T*_*c*_, these materials indicate ferroelectric properties. The Curie temperature for SBN60 is equal to 346±0.5 K so the structure is in the ferroelectric phase at the measurement temperature of 100 K. Characteristic for this family of compounds is the packing along the *z*-axis. The NbO_6_ corner-sharing octa­hedra surround three types of vacancy tunnels with penta­gonal, square and triangular shapes. The Sr^2+^ ions partially occupy two unique sites, the first one located inside the penta­gon and the second one in the square tunnels. Consequently, they are situated on the mirror plane and the inter­section of two glide planes, respectively. The site inside the penta­gonal tunnel is additionally disordered so that the same position is shared by Ba^2+^ and Sr^2+^ ions whereas another part of the Ba^2+^ ion occupies a different position (relative occupancies 0.43:0.41:0.16). One of the Nb^V^ atoms and three of the O^2−^ ions occupy general positions. The second Nb^V^ atom is located on the inter­section of the mirror planes. Two remaining O^2−^ ions are located on the same mirror plane. Only the Nb^V^ atom and one of the O^2−^ ions which is located on the mirror plane are not disordered. Each of the remaining O^2−^ ions is split between two sites, with relative occupancies of 0.52:0.48 (O^2−^ ions in general positions) and 0.64:0.36 (O^2−^ ion on the mirror plane).

## Related literature

For detailed information about the growth of the crystals, see: Lukasiewicz *et al.* (2008 ▶). For their physical properties and possible applications in photorefractive, pyroelectric and electro-optic devices, see: Neurgaonkar *et al.* (1988 ▶); Chernaya *et al.* (2003 ▶); Megumi *et al.* (1976 ▶). For SBN61 crystals, a modulation in the structure was reported, see: Schefer *et al.* (2008 ▶); Woike *et al.* (2003 ▶). The structure of SBN61 has been determined from single crystal X-ray data; the structures of three other analogues (*x* = 34, 48, 82), with no disorder present, and the influence of temperature on their unit-cell parameters has been investigated with use of the powder data, see: Podlozhenov *et al.* (2006 ▶).

## Experimental

### 

#### Crystal data

Sr_0.6_Ba_0.4_Nb_2_O_6_
*M* *_r_* = 389.33
Tetragonal, 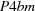

*a* = 12.43478 (12) Å
*c* = 3.93697 (6) Å
*V* = 608.75 (1) Å^3^
*Z* = 5
Mo *K*α radiation
μ = 14.32 mm^−1^
*T* = 100 K
0.06 (radius) mm

#### Data collection

Agilent Xcalibur Opal diffractometer
Absorption correction: for a sphere (*CrysAlis PRO*; Agilent, 2012 ▶) *T*_min_ = 0.278, *T*_max_ = 0.278
31766 measured reflections
2265 independent reflections
2204 reflections with *I* > 2σ(*I*)
*R*_int_ = 0.030

#### Refinement

*R*[*F* ^2^ > 2σ(*F* ^2^)] = 0.027
*wR*(*F* ^2^) = 0.074
*S* = 1.16
2265 reflections
82 parameters
15 restraints
Δρ_max_ = 2.15 e Å^−3^
Δρ_min_ = −1.96 e Å^−3^
Absolute structure: Flack parameter determined using 942 quotients [(I^+^)−(I^−^)]/[(I^+^)+(I^−^)] (Parsons & Flack, 2004 ▶)
Absolute structure parameter: 0.07 (7)

### 

Data collection: *CrysAlis PRO* (Agilent, 2012 ▶); cell refinement: *CrysAlis PRO*; data reduction: *CrysAlis PRO*; program(s) used to solve structure: *SHELXS97* (Sheldrick, 2008 ▶); program(s) used to refine structure: *SHELXL97* (Sheldrick, 2008 ▶); molecular graphics: *DIAMOND* (Brandenburg & Putz, 1999 ▶); software used to prepare material for publication: *OLEX2* (Dolomanov *et al.*, 2009 ▶).

## Supplementary Material

Crystal structure: contains datablock(s) I, global. DOI: 10.1107/S1600536813025142/ff2110sup1.cif

Structure factors: contains datablock(s) I. DOI: 10.1107/S1600536813025142/ff2110Isup2.hkl

Additional supplementary materials:  crystallographic information; 3D view; checkCIF report

## Figures and Tables

**Fig. 1.**
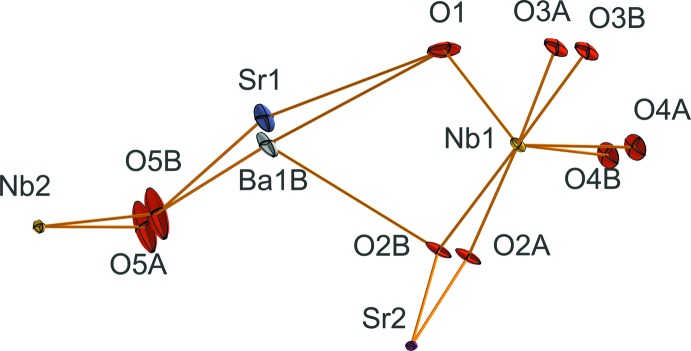
Anisotropic displacement parameters with 50% probability level of SBN60.

**Fig. 2.**
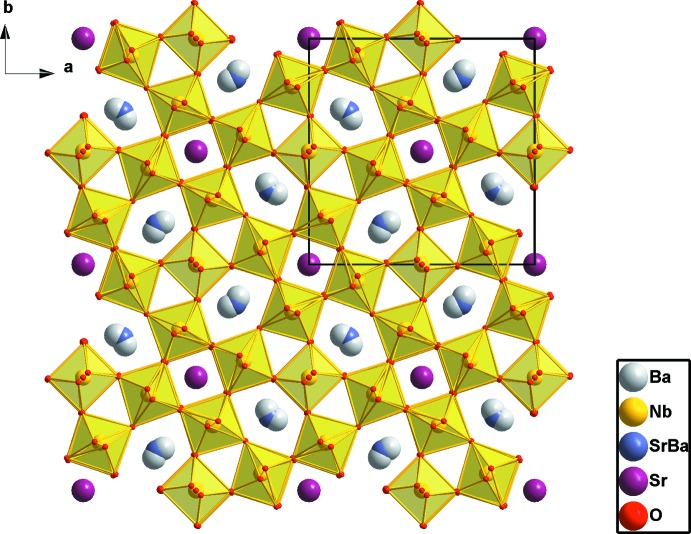
The packing of SBN60 along the Z-axis. Nb atoms are surrounded by oxygen octahedra.

## supplementary crystallographic information

### 1. Comment

Strontium-barium niobate is a very attractive material because of many applications. Its crystal structure, with open tetragonal tungsten bronze crystal lattice, exhibits a special ferroelectric-relaxor behaviour which depends on the Sr/Ba ratio (over 0.25≤*x*≤0.75). The reason to investigate SBN with different compositions is a gradual crossover from typical ferroelectric (SBN40) to extreme relaxor (SBN75). Its low-phase transition temperature, which varies from 50 to 150°C, is dependent on concentration of Sr (Megumi *et al.* 1976). Also their other properties such as: large electro-optic and pyroelectric coefficients, and good photorefractive and acousto-optic parameters (Neurgaonkar *et al.* 1988) are worth to be studied. The tetragonal structure of SBN contains a number of vacant sites which allows for introduction of a wide range of dopants including the rare earth and transition metal ions. This creates additional interesting properties. This versatility of SBN makes it a very promising material for use in optoelectronics.

The crystal structure of the title compound, Sr_0.6_Ba_0.4_Nb_2_O_6_ (SBN60) was determined at 100 K. The Curie temperature for SBN60 is equal to 346±0.5 K so the structure is in the ferroelectric phase. No modulation was observed for the complete dataset of sinΘ_max_/λ=1.06 which allowed for the refinement of the disorder of atomic positions present in the structure.

Characteristic for this family of compounds is packing along the *z*-axis. The NbO_6_ corner-sharing octahedra surround three types of vacancy tunnels with the shape of pentagon, square and triangle. The Sr ions partially occupy two unique sites, the first one located inside the pentagon and the second one in the above mentioned square-like tunnels. Consequently, they are situated on the mirror plane and the intersection of two glide planes, respectively. The site inside pentagon tunnel is additionally disordered in the way that the same position is shared by barium and strontium ion (Ba1 and Sr1) whereas another part of the barium ion (Ba1B) occupies different position. One of the niobium ions and three of the oxygen ions occupy general positions. Second niobium ion (Nb2) is located on the intersection of the mirror planes. Two remaining oxygen ions (O1, O5) are located on the same mirror plane. Only the niobium ions and one of the oxygen ions which is placed at the mirror plane (O1) are free from disorder. Each of the remaining oxygen ions is split between two positions, with relative occupancies 52:48 for O2, O3, O4, and 64:36 for O5.

### 2. Experimental

The group of single crystals with varying composition was synthetized and grown by Czochralski technique at the Institute of Electronic Materials Technology in Warsaw, Poland The detailed information about the growth of the crystals was presented elsewhere (Lukasiewicz *et al.* 2008).

The real composition of grown crystals was checked with use of ICP-OES (inductively coupled plasma-optical emission spectroscopy) method. The obtained results (in relation to niobium) are presented in wt% for corresponding oxides. SrO: 15.788; BaO: 14.983; Nb_2_O_5_: 68.465; O: 24.61. These values lead to the formula: Sr_0.59_Ba_0.38_Nb_2_O_5.97_. In the structure refinement the simplified formula Sr_0.6_Ba_0.4_Nb_2_O_6_ was used.

The single crystals of SBN60 were prepared for the X-ray experiment by grinding in a special mill to a spherical shape of *ca* 0.3–0.5 mm diameter.

### 3. Refinement

The complete X-ray diffraction data collected for the SBN60 (sinΘ_max_/λ=1.06) allowed for refinement of rich disorder of atomic positions present in this structure. The crystal was twinned by merohedry. The twinning is not directly detectable from the reflection pattern, however under measurement temperature (100 K) the compound exhibits ferroelectric properties. This excludes the presence of centres of symmetry in the studied structure. For this reason the best matching space group P4/mbm was rejected and P4bm was chosen instead.

The asymmetric part of the unit cell contains 5 independent oxygen atoms. Three of them occupy the general positions (8 d in the Wyckoff notation) and each of these three oxygen atoms appears at two positions with the ratio of occupation factors equal to 52:48 (PART 1: O2A, O3A, O4A and PART 2: O2B, O3B, O4B). Anisotropic displacement parameters (ADPs) are constrained to be equal for each pair of the disordered atoms. Remaining two oxygen atoms from the asymmetric part of the unit cell are in special positions. The one in the position 2 b in the Wyckoff notation is disordered with the 64:36 occupation factor ratio thus giving the O5A and O5B atoms. Both parts of this disorder are constrained to have equal ADPs. The last oxygen atom (O1 - position 4 c in the Wyckoff notation) does not exhibit any disorder.

In the case of heavier atoms an interesting disorder was found in the position 4 c occupied by strontium and barium atoms with the ratio of occupation factors equal to 43:41:16 (Sr1:Ba1:Ba1B, see Figure 1). The first position is occupied by Sr1 and Ba1. These two atoms are constrained to have equal ADPs whereas only Ba1B is present at the other position. Figure 2 presents the projection of the plane along the z-axis. Each Sr1/Ba1 disordered site is between two oxygen pentagons (one pentagon above, and the other one below the plane formed by Sr1/Ba1 atoms). A similar situation is present for all Sr2 positions (2 a in the Wyckoff notation) although they are surrounded by oxygen atoms forming squares. This illustration clearly shows that disorder of atoms at the 4 c Wyckoff position occupied by strontium and barium is associated with a larger free space available for these positions.

### Crystal data

**Table:**

Sr_0.6_Ba_0.4_Nb_2_O_6_	*D*_x_ = 5.310 Mg m^−^^3^
*M**_r_* = 389.33	Mo *K*α radiation, λ = 0.71073 Å
Tetragonal, *P*4*b**m*	Cell parameters from 16657 reflections
*a* = 12.43478 (12) Å	θ = 3.3–42.2°
*c* = 3.93697 (6) Å	µ = 14.32 mm^−^^1^
*V* = 608.75 (1) Å^3^	*T* = 100 K
*Z* = 5	Sphere, colourless
*F*(000) = 876	0.12 × 0.12 × 0.12 × 0.06 (radius) mm

### Data collection

**Table:**

Agilent Xcalibur Opal diffractometer	2265 independent reflections
Radiation source: fine-focus sealed tube	2204 reflections with *I* > 2σ(*I*)
Graphite monochromator	*R*_int_ = 0.030
Detector resolution: 8.4441 pixels mm^-1^	θ_max_ = 42.3°, θ_min_ = 3.3°
ω scans	*h* = −23→23
Absorption correction: for a sphere (*CrysAlis PRO*; Agilent, 2012)	*k* = −23→23
*T*_min_ = 0.278, *T*_max_ = 0.278	*l* = −7→7
31766 measured reflections	

### Refinement

**Table:**

Refinement on *F*^2^	Secondary atom site location: difference Fourier map
Least-squares matrix: full	*w* = 1/[σ^2^(*F*_o_^2^) + (0.0417*P*)^2^ + 1.9722*P*] where *P* = (*F*_o_^2^ + 2*F*_c_^2^)/3
*R*[*F*^2^ > 2σ(*F*^2^)] = 0.027	(Δ/σ)_max_ = 0.001
*wR*(*F*^2^) = 0.074	Δρ_max_ = 2.15 e Å^−^^3^
*S* = 1.16	Δρ_min_ = −1.96 e Å^−^^3^
2265 reflections	Extinction correction: *SHELXL*, Fc^*^=kFc[1+0.001xFc^2^λ^3^/sin(2θ)]^-1/4^
82 parameters	Extinction coefficient: 0.0340 (11)
15 restraints	Absolute structure: Flack parameter determined using 942 quotients [(I+)-(I-)]/[(I+)+(I-)] (Parsons & Flack, 2004)
Primary atom site location: structure-invariant direct methods	Absolute structure parameter: 0.07 (7)

### Special details

**Table:**

Experimental. Empirical absorption correction using spherical harmonics, implemented in SCALE3 ABSPACK scaling algorithm.
Geometry. All e.s.d.'s (except the e.s.d. in the dihedral angle between two l.s. planes) are estimated using the full covariance matrix. The cell e.s.d.'s are taken into account individually in the estimation of e.s.d.'s in distances, angles and torsion angles; correlations between e.s.d.'s in cell parameters are only used when they are defined by crystal symmetry. An approximate (isotropic) treatment of cell e.s.d.'s is used for estimating e.s.d.'s involving l.s. planes.
Refinement. Refinement of *F*^2^ against ALL reflections. The weighted *R*-factor *wR* and goodness of fit *S* are based on *F*^2^, conventional *R*-factors *R* are based on *F*, with *F* set to zero for negative *F*^2^. The threshold expression of *F*^2^ > σ(*F*^2^) is used only for calculating *R*-factors(gt) *etc*. and is not relevant to the choice of reflections for refinement. *R*-factors based on *F*^2^ are statistically about twice as large as those based on *F*, and *R*- factors based on ALL data will be even larger.

### Fractional atomic coordinates and isotropic or equivalent isotropic displacement parameters (Å^2^)

**Table:**

	*x*	*y*	*z*	*U*_iso_*/*U*_eq_	Occ. (<1)
Ba1	0.82718 (6)	0.32718 (6)	0.5075 (5)	0.01003 (15)	0.36
Ba1B	0.8140 (6)	0.3543 (3)	0.507 (2)	0.0104 (7)	0.07
Nb1	0.574560 (15)	0.288626 (15)	0.00050 (15)	0.00455 (6)	
Nb2	1.0000	0.5000	0.98772 (12)	0.00311 (7)	
Sr1	0.82718 (6)	0.32718 (6)	0.5075 (5)	0.01003 (15)	0.38
Sr2	0.5000	0.5000	0.5120 (2)	0.00246 (9)	0.74
O1	0.7187 (2)	0.2187 (2)	0.0349 (15)	0.0144 (8)	
O2A	0.6341 (5)	0.4334 (4)	−0.0139 (19)	0.0081 (6)	0.48
O2B	0.6437 (5)	0.4286 (4)	0.0985 (14)	0.0081 (6)	0.52
O3A	0.4966 (5)	0.1552 (4)	0.1035 (15)	0.0085 (6)	0.48
O3B	0.4907 (3)	0.1577 (3)	−0.0208 (16)	0.0085 (6)	0.52
O4A	0.5510 (5)	0.2845 (4)	−0.4624 (11)	0.0121 (6)	0.48
O4B	0.5974 (4)	0.3066 (4)	−0.4656 (12)	0.0121 (6)	0.52
O5A	1.0000	0.5000	0.5179 (3)	0.027 (3)	0.36
O5B	0.9789 (8)	0.4789 (8)	0.5274 (7)	0.027 (3)	0.32

### Atomic displacement parameters (Å^2^)

**Table:**

	*U*^11^	*U*^22^	*U*^33^	*U*^12^	*U*^13^	*U*^23^
Ba1	0.0104 (2)	0.0104 (2)	0.00932 (18)	−0.0083 (4)	0.0002 (3)	0.0002 (3)
Ba1B	0.0170 (18)	0.0070 (12)	0.0071 (9)	−0.0079 (15)	0.000 (2)	0.000 (2)
Nb1	0.00466 (8)	0.00369 (8)	0.00531 (8)	−0.00120 (5)	−0.00007 (12)	−0.00130 (12)
Nb2	0.00313 (9)	0.00313 (9)	0.00306 (14)	−0.00024 (9)	0.000	0.000
Sr1	0.0104 (2)	0.0104 (2)	0.00932 (18)	−0.0083 (4)	0.0002 (3)	0.0002 (3)
Sr2	0.00182 (11)	0.00182 (11)	0.0037 (2)	0.000	0.000	0.000
O1	0.0067 (6)	0.0067 (6)	0.030 (3)	0.0034 (7)	0.0001 (11)	0.0001 (11)
O2A	0.0107 (11)	0.0029 (8)	0.011 (2)	−0.0036 (7)	0.0031 (16)	−0.0024 (14)
O2B	0.0107 (11)	0.0029 (8)	0.011 (2)	−0.0036 (7)	0.0031 (16)	−0.0024 (14)
O3A	0.0079 (8)	0.0047 (7)	0.0130 (17)	−0.0039 (6)	0.0006 (14)	−0.0017 (15)
O3B	0.0079 (8)	0.0047 (7)	0.0130 (17)	−0.0039 (6)	0.0006 (14)	−0.0017 (15)
O4A	0.0233 (16)	0.0104 (12)	0.0026 (14)	−0.0029 (10)	0.0000 (13)	0.0016 (10)
O4B	0.0233 (16)	0.0104 (12)	0.0026 (14)	−0.0029 (10)	0.0000 (13)	0.0016 (10)
O5A	0.038 (5)	0.038 (5)	0.005 (3)	−0.026 (6)	0.000	0.000
O5B	0.038 (5)	0.038 (5)	0.005 (3)	−0.026 (6)	0.000	0.000

### Geometric parameters (Å, º)

**Table:**

Ba1—Nb2	3.5792 (15)	Nb2—O5A^i^	2.0872 (10)
Ba1—O1^i^	2.820 (5)	Nb2—O5A	1.8498 (10)
Ba1—O1	2.665 (5)	Nb2—O5B^xii^	1.8496 (10)
Ba1—O2A^ii^	3.326 (6)	Nb2—O5B	1.8496 (10)
Ba1—O2A^i^	3.326 (6)	Sr2—O2A^i^	2.636 (7)
Ba1—O3A^iii^	3.161 (6)	Sr2—O2A^iv^	2.636 (7)
Ba1—O3A^iv^	3.161 (6)	Sr2—O2A^xiii^	2.636 (7)
Ba1—O3A^v^	2.649 (6)	Sr2—O2A^xiv^	2.636 (7)
Ba1—O3A^vi^	2.649 (6)	Sr2—O2B^ix^	2.576 (6)
Ba1—O4A^iv^	3.112 (6)	Sr2—O2B^xv^	2.576 (6)
Ba1—O4A^iii^	3.112 (6)	Sr2—O2B	2.576 (6)
Ba1—O5A	3.0395 (11)	Sr2—O2B^vi^	2.576 (6)
Ba1B—Ba1B^vii^	3.9370	Sr2—O4B^xiii^	2.694 (5)
Ba1B—Ba1B^i^	3.9370	Sr2—O4B^i^	2.694 (5)
Ba1B—Ba1B^viii^	0.709 (11)	Sr2—O4B^iv^	2.694 (5)
Ba1B—Nb1^i^	3.648 (8)	Sr2—O4B^xiv^	2.694 (5)
Ba1B—Nb1	3.675 (8)	O1—Ba1^vii^	2.820 (5)
Ba1B—Nb1^ii^	3.997 (5)	O1—Ba1B^xvi^	2.927 (9)
Ba1B—Nb1^vi^	3.620 (6)	O1—Ba1B^vii^	2.927 (9)
Ba1B—Nb1^iv^	3.592 (6)	O1—Ba1B^viii^	2.775 (8)
Ba1B—Nb2	3.495 (7)	O1—Nb1^viii^	1.9967 (12)
Ba1B—Nb2^vii^	3.579 (8)	O1—Sr1^vii^	2.820 (5)
Ba1B—O1	2.775 (8)	O2A—Ba1^vii^	3.326 (6)
Ba1B—O1^i^	2.927 (9)	O2A—Nb1^vi^	2.002 (5)
Nb1—Sr2^vii^	3.3862 (6)	O2A—Sr2^vii^	2.636 (7)
Nb1—O1	1.9966 (12)	O2B—Nb1^vi^	2.038 (5)
Nb1—O2A^ix^	2.002 (5)	O2B—Sr2^vii^	3.052 (5)
Nb1—O2A	1.948 (5)	O3A—Ba1^xvii^	3.161 (6)
Nb1—O2B^ix^	2.038 (5)	O3A—Ba1^ix^	2.649 (6)
Nb1—O2B	1.979 (5)	O3A—Nb2^xviii^	1.983 (5)
Nb1—O3A	1.964 (5)	O3B—Nb2^xviii^	1.965 (4)
Nb1—O3B	1.935 (4)	O3B—Sr1^ix^	2.915 (5)
Nb1—O4A	1.846 (5)	O3B—Sr1^xvii^	2.760 (6)
Nb1—O4A^i^	2.135 (4)	O4A—Ba1^xvii^	3.112 (6)
Nb1—O4B	1.870 (5)	O4A—Nb1^vii^	2.135 (4)
Nb1—O4B^i^	2.133 (4)	O4A—Sr2^vii^	2.755 (5)
Nb2—O3A^x^	1.983 (5)	O4B—Nb1^vii^	2.133 (4)
Nb2—O3A^xi^	1.983 (5)	O4B—Sr1^vii^	2.870 (6)
Nb2—O3A^iii^	1.983 (5)	O4B—Sr2^vii^	2.694 (5)
Nb2—O3A^iv^	1.983 (5)	O5A—Ba1^xii^	3.0395 (11)
Nb2—O3B^iii^	1.965 (4)	O5A—Nb2^vii^	2.0871 (10)
Nb2—O3B^iv^	1.965 (4)	O5B—Nb2^vii^	2.157 (5)
Nb2—O3B^xi^	1.965 (4)	O5B—O5B^xii^	0.74 (3)
Nb2—O3B^x^	1.965 (4)		
			
O1^i^—Ba1—Nb2	100.70 (12)	O3A^x^—Nb2—O3A^iii^	89.4 (3)
O1—Ba1—Nb2	167.60 (13)	O3A^x^—Nb2—O5A^i^	76.71 (17)
O1—Ba1—O1^i^	91.70 (11)	O3A^iii^—Nb2—O5A^i^	76.71 (17)
O1—Ba1—O2A^i^	103.37 (12)	O3A^iv^—Nb2—O5A^i^	76.71 (17)
O1^i^—Ba1—O2A^i^	55.08 (11)	O3A^xi^—Nb2—O5A^i^	76.71 (17)
O1—Ba1—O2A^ii^	103.37 (12)	O3B^xi^—Nb2—O3A^x^	85.71 (16)
O1^i^—Ba1—O2A^ii^	55.08 (11)	O3B^x^—Nb2—O3A^x^	14.4 (2)
O1^i^—Ba1—O3A^iii^	78.78 (15)	O3B^iv^—Nb2—O3A^x^	167.5 (3)
O1—Ba1—O3A^iii^	152.97 (11)	O3B^iii^—Nb2—O3A^x^	94.74 (16)
O1—Ba1—O3A^iv^	152.97 (11)	O3B^x^—Nb2—O3A^xi^	85.71 (16)
O1^i^—Ba1—O3A^iv^	78.78 (15)	O3B^iv^—Nb2—O3A^xi^	94.74 (16)
O1^i^—Ba1—O4A^iv^	100.74 (10)	O3B^iii^—Nb2—O3A^xi^	167.5 (3)
O1—Ba1—O4A^iv^	104.68 (10)	O3B^xi^—Nb2—O3A^xi^	14.4 (2)
O1^i^—Ba1—O4A^iii^	100.74 (10)	O3B^iii^—Nb2—O3A^iv^	85.71 (16)
O1—Ba1—O4A^iii^	104.68 (10)	O3B^xi^—Nb2—O3A^iv^	94.74 (16)
O1—Ba1—O5A	136.48 (14)	O3B^x^—Nb2—O3A^iv^	167.5 (3)
O1^i^—Ba1—O5A	131.81 (13)	O3B^iv^—Nb2—O3A^iv^	14.4 (2)
O2A^ii^—Ba1—Nb2	84.01 (10)	O3B^iii^—Nb2—O3A^iii^	14.4 (2)
O2A^i^—Ba1—Nb2	84.01 (11)	O3B^xi^—Nb2—O3A^iii^	167.5 (3)
O2A^ii^—Ba1—O2A^i^	104.6 (2)	O3B^x^—Nb2—O3A^iii^	94.74 (16)
O3A^v^—Ba1—Nb2	77.88 (13)	O3B^iv^—Nb2—O3A^iii^	85.71 (16)
O3A^iii^—Ba1—Nb2	33.50 (10)	O3B^x^—Nb2—O3B^iv^	178.0 (4)
O3A^vi^—Ba1—Nb2	77.88 (13)	O3B^xi^—Nb2—O3B^iv^	96.7 (2)
O3A^iv^—Ba1—Nb2	33.50 (10)	O3B^iii^—Nb2—O3B^iv^	83.2 (3)
O3A^vi^—Ba1—O1^i^	149.58 (12)	O3B^iii^—Nb2—O3B^x^	96.7 (2)
O3A^v^—Ba1—O1^i^	149.58 (11)	O3B^xi^—Nb2—O3B^x^	83.2 (3)
O3A^vi^—Ba1—O1	91.44 (17)	O3B^iii^—Nb2—O3B^xi^	178.0 (4)
O3A^v^—Ba1—O1	91.44 (17)	O3B^x^—Nb2—O5A^i^	90.98 (18)
O3A^v^—Ba1—O2A^i^	151.82 (14)	O3B^iii^—Nb2—O5A^i^	90.98 (18)
O3A^vi^—Ba1—O2A^i^	94.82 (16)	O3B^iv^—Nb2—O5A^i^	90.98 (18)
O3A^v^—Ba1—O2A^ii^	94.82 (16)	O3B^xi^—Nb2—O5A^i^	90.98 (18)
O3A^vi^—Ba1—O2A^ii^	151.82 (15)	O5A—Nb2—O3A^xi^	103.29 (17)
O3A^iv^—Ba1—O2A^i^	50.56 (14)	O5A—Nb2—O3A^x^	103.29 (17)
O3A^iii^—Ba1—O2A^i^	91.89 (17)	O5A—Nb2—O3A^iii^	103.29 (17)
O3A^iii^—Ba1—O2A^ii^	50.56 (14)	O5A—Nb2—O3A^iv^	103.29 (17)
O3A^iv^—Ba1—O2A^ii^	91.89 (17)	O5A—Nb2—O3B^xi^	89.02 (18)
O3A^v^—Ba1—O3A^vi^	60.5 (2)	O5A—Nb2—O3B^x^	89.02 (18)
O3A^v^—Ba1—O3A^iv^	109.6 (2)	O5A—Nb2—O3B^iv^	89.02 (18)
O3A^vi^—Ba1—O3A^iii^	109.6 (2)	O5A—Nb2—O3B^iii^	89.02 (18)
O3A^v^—Ba1—O3A^iii^	84.84 (16)	O5A—Nb2—O5A^i^	180.000 (1)
O3A^vi^—Ba1—O3A^iv^	84.84 (16)	O5B—Nb2—O3A^x^	111.5 (4)
O3A^iii^—Ba1—O3A^iv^	49.95 (19)	O5B^xii^—Nb2—O3A^x^	94.8 (4)
O3A^vi^—Ba1—O4A^iii^	107.68 (15)	O5B—Nb2—O3A^xi^	111.5 (4)
O3A^vi^—Ba1—O4A^iv^	49.36 (15)	O5B^xii^—Nb2—O3A^xi^	94.8 (4)
O3A^v^—Ba1—O4A^iv^	107.68 (15)	O5B^xii^—Nb2—O3A^iii^	111.5 (4)
O3A^v^—Ba1—O4A^iii^	49.36 (15)	O5B^xii^—Nb2—O3A^iv^	111.5 (4)
O3A^v^—Ba1—O5A	52.21 (13)	O5B—Nb2—O3A^iv^	94.8 (4)
O3A^vi^—Ba1—O5A	52.21 (13)	O5B—Nb2—O3A^iii^	94.8 (4)
O4A^iii^—Ba1—Nb2	73.20 (10)	O5B^xii^—Nb2—O3B^iii^	97.6 (4)
O4A^iv^—Ba1—Nb2	73.20 (10)	O5B—Nb2—O3B^iii^	80.4 (4)
O4A^iv^—Ba1—O2A^i^	45.66 (14)	O5B^xii^—Nb2—O3B^x^	80.4 (4)
O4A^iv^—Ba1—O2A^ii^	143.25 (16)	O5B—Nb2—O3B^x^	97.6 (4)
O4A^iii^—Ba1—O2A^ii^	45.66 (14)	O5B^xii^—Nb2—O3B^iv^	97.6 (4)
O4A^iii^—Ba1—O2A^i^	143.25 (16)	O5B—Nb2—O3B^iv^	80.4 (4)
O4A^iii^—Ba1—O3A^iv^	101.95 (14)	O5B^xii^—Nb2—O3B^xi^	80.4 (4)
O4A^iv^—Ba1—O3A^iii^	101.95 (14)	O5B—Nb2—O3B^xi^	97.6 (4)
O4A^iii^—Ba1—O3A^iii^	53.62 (13)	O5B^xii^—Nb2—O5A	11.6 (4)
O4A^iv^—Ba1—O3A^iv^	53.62 (13)	O5B—Nb2—O5A	11.6 (4)
O4A^iii^—Ba1—O4A^iv^	142.8 (2)	O5B—Nb2—O5A^i^	168.4 (4)
O5A—Ba1—Nb2	31.12 (2)	O5B^xii^—Nb2—O5A^i^	168.4 (4)
O5A—Ba1—O2A^ii^	102.82 (9)	O5B^xii^—Nb2—O5B	23.1 (9)
O5A—Ba1—O2A^i^	102.82 (9)	O2A^i^—Sr2—O2A^xiv^	59.91 (16)
O5A—Ba1—O3A^iii^	57.98 (11)	O2A^xiii^—Sr2—O2A^xiv^	59.91 (16)
O5A—Ba1—O3A^iv^	57.98 (11)	O2A^iv^—Sr2—O2A^i^	59.91 (16)
O5A—Ba1—O4A^iii^	71.50 (10)	O2A^iv^—Sr2—O2A^xiv^	89.8 (3)
O5A—Ba1—O4A^iv^	71.50 (10)	O2A^i^—Sr2—O2A^xiii^	89.8 (3)
Ba1B^viii^—Ba1B—Ba1B^i^	90.001 (1)	O2A^iv^—Sr2—O2A^xiii^	59.91 (16)
Ba1B^viii^—Ba1B—Ba1B^vii^	90.0	O2A^iv^—Sr2—O4B^i^	113.59 (15)
Ba1B^i^—Ba1B—Ba1B^vii^	179.999 (3)	O2A^iv^—Sr2—O4B^iv^	54.00 (15)
Ba1B^viii^—Ba1B—Nb1	114.56 (7)	O2A^i^—Sr2—O4B^i^	54.00 (15)
Ba1B^viii^—Ba1B—Nb1^iv^	142.21 (13)	O2A^xiii^—Sr2—O4B^i^	122.79 (15)
Ba1B^viii^—Ba1B—Nb1^ii^	55.97 (11)	O2A^xiv^—Sr2—O4B^i^	63.48 (16)
Ba1B^viii^—Ba1B—Nb1^vi^	141.65 (13)	O2A^xiv^—Sr2—O4B^xiv^	54.00 (15)
Ba1B^viii^—Ba1B—Nb1^i^	114.75 (7)	O2A^xiv^—Sr2—O4B^xiii^	113.59 (15)
Ba1B^vii^—Ba1B—Nb1^ii^	119.09 (12)	O2A^xiii^—Sr2—O4B^xiii^	54.00 (15)
Ba1B^i^—Ba1B—Nb1^ii^	60.91 (12)	O2A^i^—Sr2—O4B^xiii^	122.79 (15)
Ba1B^viii^—Ba1B—Nb2	84.18 (9)	O2A^iv^—Sr2—O4B^xiii^	63.48 (16)
Ba1B^viii^—Ba1B—Nb2^vii^	84.32 (8)	O2A^xiii^—Sr2—O4B^xiv^	63.48 (16)
Ba1B^viii^—Ba1B—O1^i^	83.04 (12)	O2A^i^—Sr2—O4B^iv^	63.48 (16)
Ba1B^viii^—Ba1B—O1	82.66 (13)	O2A^xiii^—Sr2—O4B^iv^	113.59 (15)
Nb1^i^—Ba1B—Ba1B^i^	57.81 (13)	O2A^xiv^—Sr2—O4B^iv^	122.79 (16)
Nb1^iv^—Ba1B—Ba1B^i^	57.25 (13)	O2A^iv^—Sr2—O4B^xiv^	122.79 (16)
Nb1^vi^—Ba1B—Ba1B^i^	123.42 (13)	O2A^i^—Sr2—O4B^xiv^	113.59 (15)
Nb1—Ba1B—Ba1B^i^	122.85 (14)	O2B—Sr2—O2A^xiv^	116.58 (18)
Nb1^iv^—Ba1B—Ba1B^vii^	122.75 (13)	O2B^vi^—Sr2—O2A^xiv^	174.1 (2)
Nb1^i^—Ba1B—Ba1B^vii^	122.19 (13)	O2B^ix^—Sr2—O2A^i^	116.61 (17)
Nb1—Ba1B—Ba1B^vii^	57.15 (14)	O2B^xv^—Sr2—O2A^i^	174.1 (2)
Nb1^vi^—Ba1B—Ba1B^vii^	56.58 (13)	O2B^vi^—Sr2—O2A^i^	116.58 (18)
Nb1^vi^—Ba1B—Nb1^ii^	154.0 (2)	O2B—Sr2—O2A^iv^	116.61 (17)
Nb1^vi^—Ba1B—Nb1^i^	100.09 (17)	O2B^ix^—Sr2—O2A^iv^	174.1 (2)
Nb1^iv^—Ba1B—Nb1	100.09 (17)	O2B^xv^—Sr2—O2A^iv^	116.58 (18)
Nb1—Ba1B—Nb1^ii^	90.37 (11)	O2B^vi^—Sr2—O2A^iv^	84.29 (16)
Nb1^i^—Ba1B—Nb1^ii^	58.79 (10)	O2B—Sr2—O2A^xiii^	174.1 (2)
Nb1^i^—Ba1B—Nb1	65.04 (12)	O2B^ix^—Sr2—O2A^xiv^	84.29 (16)
Nb1^iv^—Ba1B—Nb1^vi^	66.17 (7)	O2B^xv^—Sr2—O2A^xiv^	116.61 (17)
Nb1^vi^—Ba1B—Nb1	65.41 (13)	O2B^vi^—Sr2—O2A^xiii^	116.61 (17)
Nb1^iv^—Ba1B—Nb1^ii^	111.9 (2)	O2B—Sr2—O2A^i^	84.29 (16)
Nb1^iv^—Ba1B—Nb1^i^	65.96 (14)	O2B^ix^—Sr2—O2A^xiii^	116.58 (18)
Nb2—Ba1B—Ba1B^i^	57.21 (14)	O2B^xv^—Sr2—O2A^xiii^	84.29 (16)
Nb2^vii^—Ba1B—Ba1B^vii^	55.18 (13)	O2B^xv^—Sr2—O2B	101.6 (2)
Nb2—Ba1B—Ba1B^vii^	122.79 (14)	O2B^ix^—Sr2—O2B	66.44 (13)
Nb2^vii^—Ba1B—Ba1B^i^	124.83 (13)	O2B^xv^—Sr2—O2B^vi^	66.44 (13)
Nb2—Ba1B—Nb1^vi^	98.01 (8)	O2B^ix^—Sr2—O2B^vi^	101.6 (2)
Nb2^vii^—Ba1B—Nb1^vi^	61.99 (11)	O2B^ix^—Sr2—O2B^xv^	66.44 (13)
Nb2—Ba1B—Nb1^i^	111.6 (2)	O2B^vi^—Sr2—O2B	66.44 (13)
Nb2^vii^—Ba1B—Nb1^iv^	98.78 (8)	O2B^ix^—Sr2—O4B^i^	63.67 (15)
Nb2^vii^—Ba1B—Nb1^ii^	140.28 (19)	O2B^xv^—Sr2—O4B^i^	129.83 (15)
Nb2—Ba1B—Nb1^ii^	103.9 (2)	O2B^vi^—Sr2—O4B^i^	119.00 (16)
Nb2—Ba1B—Nb1^iv^	63.06 (11)	O2B—Sr2—O4B^i^	53.19 (16)
Nb2^vii^—Ba1B—Nb1^i^	160.91 (15)	O2B—Sr2—O4B^xiii^	129.83 (15)
Nb2^vii^—Ba1B—Nb1	109.0 (2)	O2B^ix^—Sr2—O4B^xiv^	53.19 (16)
Nb2—Ba1B—Nb1	160.94 (14)	O2B^xv^—Sr2—O4B^xiv^	63.67 (15)
Nb2—Ba1B—Nb2^vii^	67.62 (11)	O2B^vi^—Sr2—O4B^xiv^	129.83 (15)
O1^i^—Ba1B—Ba1B^i^	44.76 (17)	O2B—Sr2—O4B^iv^	63.67 (15)
O1—Ba1B—Ba1B^i^	132.0 (2)	O2B^ix^—Sr2—O4B^iv^	129.83 (15)
O1^i^—Ba1B—Ba1B^vii^	135.24 (17)	O2B^xv^—Sr2—O4B^iv^	119.00 (16)
O1—Ba1B—Ba1B^vii^	48.0 (2)	O2B^vi^—Sr2—O4B^iv^	53.19 (16)
O1^i^—Ba1B—Nb1^iv^	84.9 (2)	O2B^ix^—Sr2—O4B^xiii^	119.00 (16)
O1—Ba1B—Nb1^ii^	76.29 (12)	O2B^xv^—Sr2—O4B^xiii^	53.19 (16)
O1^i^—Ba1B—Nb1^ii^	28.53 (5)	O2B^vi^—Sr2—O4B^xiii^	63.67 (15)
O1—Ba1B—Nb1^iv^	132.4 (2)	O2B—Sr2—O4B^xiv^	119.00 (16)
O1^i^—Ba1B—Nb1^vi^	133.2 (2)	O4B^xiii^—Sr2—O4B^i^	176.3 (2)
O1^i^—Ba1B—Nb1^i^	33.11 (9)	O4B^xiv^—Sr2—O4B^i^	89.939 (7)
O1^i^—Ba1B—Nb1	85.94 (18)	O4B^iv^—Sr2—O4B^i^	89.939 (8)
O1—Ba1B—Nb1^i^	82.66 (19)	O4B^xiv^—Sr2—O4B^xiii^	89.939 (7)
O1—Ba1B—Nb1	32.41 (9)	O4B^iv^—Sr2—O4B^xiii^	89.939 (7)
O1—Ba1B—Nb1^vi^	86.6 (2)	O4B^iv^—Sr2—O4B^xiv^	176.3 (2)
O1—Ba1B—Nb2	163.8 (3)	Ba1—O1—Ba1^vii^	91.70 (11)
O1^i^—Ba1B—Nb2	100.5 (3)	Ba1—O1—Ba1B^xvi^	89.96 (19)
O1—Ba1B—Nb2^vii^	101.6 (3)	Ba1—O1—Ba1B^vii^	89.96 (19)
O1^i^—Ba1B—Nb2^vii^	163.5 (3)	Ba1—O1—Sr1^vii^	91.70 (11)
O1—Ba1B—O1^i^	87.28 (17)	Ba1B^viii^—O1—Ba1^vii^	89.9 (2)
O1—Nb1—Sr2^vii^	128.49 (14)	Ba1B—O1—Ba1^vii^	89.9 (2)
O1—Nb1—O2A^ix^	176.4 (2)	Ba1B—O1—Ba1B^xvi^	89.1 (2)
O1—Nb1—O2B^ix^	165.2 (2)	Ba1B^viii^—O1—Ba1B^xvi^	87.28 (17)
O1—Nb1—O4A^i^	92.6 (2)	Ba1B—O1—Ba1B^vii^	87.28 (17)
O1—Nb1—O4B^i^	81.9 (2)	Ba1B^viii^—O1—Ba1B^vii^	89.1 (2)
O2A—Nb1—Sr2^vii^	50.9 (2)	Ba1B^viii^—O1—Sr1^vii^	89.9 (2)
O2A^ix^—Nb1—Sr2^vii^	51.0 (2)	Ba1B—O1—Sr1^vii^	89.9 (2)
O2A—Nb1—O1	93.65 (19)	Nb1^viii^—O1—Ba1	106.33 (15)
O2A—Nb1—O2A^ix^	83.6 (3)	Nb1—O1—Ba1^vii^	99.86 (16)
O2A—Nb1—O2B	13.5 (2)	Nb1^viii^—O1—Ba1^vii^	99.86 (16)
O2A—Nb1—O2B^ix^	87.83 (17)	Nb1—O1—Ba1	106.33 (15)
O2A^ix^—Nb1—O2B^ix^	13.1 (2)	Nb1^viii^—O1—Ba1B^vii^	107.0 (2)
O2A—Nb1—O3A	167.1 (3)	Nb1—O1—Ba1B^vii^	93.69 (19)
O2A^ix^—Nb1—O4A^i^	85.4 (3)	Nb1—O1—Ba1B	99.43 (19)
O2A—Nb1—O4A^i^	95.9 (3)	Nb1^viii^—O1—Ba1B	113.88 (19)
O2A—Nb1—O4B^i^	83.2 (3)	Nb1^viii^—O1—Ba1B^viii^	99.43 (19)
O2A^ix^—Nb1—O4B^i^	95.4 (3)	Nb1—O1—Ba1B^viii^	113.88 (19)
O2B^ix^—Nb1—Sr2^vii^	62.81 (15)	Nb1^viii^—O1—Ba1B^xvi^	93.69 (19)
O2B—Nb1—Sr2^vii^	63.07 (16)	Nb1—O1—Ba1B^xvi^	107.0 (2)
O2B—Nb1—O1	88.8 (2)	Nb1—O1—Nb1^viii^	141.0 (2)
O2B—Nb1—O2A^ix^	87.94 (17)	Nb1—O1—Sr1^vii^	99.86 (16)
O2B—Nb1—O2B^ix^	89.3 (4)	Nb1^viii^—O1—Sr1^vii^	99.86 (16)
O2B—Nb1—O4A^i^	83.6 (2)	Nb1^vi^—O2A—Ba1^vii^	91.01 (19)
O2B^ix^—Nb1—O4A^i^	72.6 (2)	Nb1—O2A—Ba1^vii^	85.61 (18)
O2B^ix^—Nb1—O4B^i^	83.6 (2)	Nb1—O2A—Nb1^vi^	172.8 (4)
O2B—Nb1—O4B^i^	70.0 (2)	Nb1^vi^—O2A—Sr2	90.3 (2)
O3A—Nb1—Sr2^vii^	129.63 (17)	Nb1—O2A—Sr2^vii^	94.1 (2)
O3A—Nb1—O1	93.5 (2)	Nb1^vi^—O2A—Sr2^vii^	92.8 (2)
O3A—Nb1—O2A^ix^	88.8 (2)	Nb1—O2A—Sr2	91.5 (2)
O3A—Nb1—O2B^ix^	82.5 (2)	Sr2—O2A—Ba1^vii^	166.3 (3)
O3A—Nb1—O2B	156.5 (2)	Sr2^vii^—O2A—Ba1^vii^	100.4 (2)
O3A—Nb1—O4A^i^	73.0 (2)	Sr2^vii^—O2A—Sr2	93.13 (17)
O3A—Nb1—O4B^i^	87.2 (2)	Nb1—O2B—Nb1^vi^	157.8 (3)
O3B—Nb1—Sr2^vii^	118.74 (17)	Nb1^vi^—O2B—Sr2^vii^	80.75 (16)
O3B—Nb1—O1	96.90 (17)	Nb1—O2B—Sr2^vii^	81.61 (17)
O3B—Nb1—O2A^ix^	86.0 (2)	Nb1^vi^—O2B—Sr2	95.7 (2)
O3B—Nb1—O2A	168.9 (2)	Nb1—O2B—Sr2	97.2 (2)
O3B—Nb1—O2B^ix^	82.9 (2)	Sr2—O2B—Sr2^vii^	88.37 (17)
O3B—Nb1—O2B	169.2 (2)	Ba1^ix^—O3A—Ba1^xvii^	84.84 (16)
O3B—Nb1—O3A	14.6 (2)	Nb1—O3A—Ba1^xvii^	96.7 (2)
O3B—Nb1—O4A^i^	87.1 (2)	Nb1—O3A—Ba1^ix^	116.5 (3)
O3B—Nb1—O4B^i^	101.7 (2)	Nb1—O3A—Nb2^xviii^	139.8 (3)
O4A^i^—Nb1—Sr2^vii^	122.92 (14)	Nb2^xviii^—O3A—Ba1^xvii^	84.90 (18)
O4A—Nb1—Sr2^vii^	54.38 (16)	Nb2^xviii^—O3A—Ba1^ix^	103.6 (2)
O4A—Nb1—O1	101.4 (3)	Nb1—O3B—Nb2^xviii^	143.8 (3)
O4A—Nb1—O2A	93.3 (3)	Nb1—O3B—Sr1^ix^	106.9 (2)
O4A—Nb1—O2A^ix^	81.2 (3)	Nb1—O3B—Sr1^xvii^	111.6 (2)
O4A—Nb1—O2B	106.6 (2)	Nb2^xviii^—O3B—Sr1^xvii^	97.06 (18)
O4A—Nb1—O2B^ix^	93.3 (2)	Nb2^xviii^—O3B—Sr1^ix^	95.35 (18)
O4A—Nb1—O3A	95.9 (2)	Sr1^xvii^—O3B—Sr1^ix^	87.81 (12)
O4A—Nb1—O3B	81.3 (2)	Nb1—O4A—Ba1^xvii^	101.1 (2)
O4A—Nb1—O4A^i^	162.7 (4)	Nb1^vii^—O4A—Ba1^xvii^	95.49 (19)
O4A—Nb1—O4B	19.8 (2)	Nb1—O4A—Nb1^vii^	162.7 (4)
O4A—Nb1—O4B^i^	175.3 (2)	Nb1^vii^—O4A—Sr2^vii^	88.40 (16)
O4B—Nb1—Sr2^vii^	52.52 (15)	Nb1—O4A—Sr2^vii^	92.61 (19)
O4B^i^—Nb1—Sr2^vii^	120.98 (13)	Sr2^vii^—O4A—Ba1^xvii^	103.11 (19)
O4B—Nb1—O1	89.0 (2)	Nb1—O4B—Nb1^vii^	159.1 (3)
O4B—Nb1—O2A	78.6 (3)	Nb1—O4B—Sr1^vii^	101.4 (2)
O4B—Nb1—O2A^ix^	92.7 (3)	Nb1^vii^—O4B—Sr1^vii^	96.07 (19)
O4B—Nb1—O2B^ix^	105.8 (2)	Nb1^vii^—O4B—Sr2^vii^	90.09 (16)
O4B—Nb1—O2B	91.1 (2)	Nb1—O4B—Sr2^vii^	94.05 (19)
O4B—Nb1—O3A	112.3 (2)	Sr2^vii^—O4B—Sr1^vii^	111.53 (17)
O4B—Nb1—O3B	98.1 (2)	Ba1—O5A—Ba1^xii^	178.46 (10)
O4B^i^—Nb1—O4A^i^	17.2 (2)	Nb2—O5A—Ba1^xii^	90.77 (5)
O4B—Nb1—O4A^i^	174.4 (2)	Nb2^vii^—O5A—Ba1^xii^	89.23 (5)
O4B—Nb1—O4B^i^	159.1 (3)	Nb2^vii^—O5A—Ba1	89.23 (5)
O3A^xi^—Nb2—O3A^iv^	89.4 (3)	Nb2—O5A—Ba1	90.77 (5)
O3A^iii^—Nb2—O3A^iv^	84.6 (3)	Nb2—O5A—Nb2^vii^	180.0
O3A^x^—Nb2—O3A^iv^	153.4 (3)	Nb2—O5B—Nb2^vii^	158.6 (8)
O3A^xi^—Nb2—O3A^x^	84.6 (3)	O5B^xii^—O5B—Nb2^vii^	80.1 (4)
O3A^xi^—Nb2—O3A^iii^	153.4 (3)	O5B^xii^—O5B—Nb2	78.4 (4)
			
Ba1B^i^—Ba1B—O1—Ba1	−69.4 (15)	O2B^vi^—Sr2—O2A—Nb1	−155.5 (3)
Ba1B^vii^—Ba1B—O1—Ba1	110.6 (15)	O2B^ix^—Sr2—O2A—Sr2^vii^	61.38 (19)
Ba1B^viii^—Ba1B—O1—Ba1^vii^	−89.99 (3)	O2B—Sr2—O2A—Sr2^vii^	179.9 (12)
Ba1B^viii^—Ba1B—O1—Ba1	14.0 (16)	O2B^vi^—Sr2—O2A—Sr2^vii^	−61.40 (19)
Ba1B^vii^—Ba1B—O1—Ba1^vii^	6.69 (12)	O2B^xv^—Sr2—O2A—Sr2^vii^	−0.02 (19)
Ba1B^i^—Ba1B—O1—Ba1^vii^	−173.32 (12)	O2B^ix^—Sr2—O2B—Nb1^vi^	138.24 (11)
Ba1B^viii^—Ba1B—O1—Ba1B^vii^	−96.67 (13)	O2B^xv^—Sr2—O2B—Nb1^vi^	80.54 (17)
Ba1B^vii^—Ba1B—O1—Ba1B^xvi^	13.8 (2)	O2B^vi^—Sr2—O2B—Nb1^vi^	22.8 (2)
Ba1B^i^—Ba1B—O1—Ba1B^xvi^	−166.2 (2)	O2B^vi^—Sr2—O2B—Nb1	−139.03 (12)
Ba1B^viii^—Ba1B—O1—Ba1B^xvi^	−82.86 (11)	O2B^ix^—Sr2—O2B—Nb1	−23.6 (2)
Ba1B^i^—Ba1B—O1—Ba1B^vii^	180.0	O2B^xv^—Sr2—O2B—Nb1	−81.33 (18)
Ba1B^i^—Ba1B—O1—Ba1B^viii^	−83.33 (13)	O2B^vi^—Sr2—O2B—Sr2^vii^	−57.70 (7)
Ba1B^vii^—Ba1B—O1—Ba1B^viii^	96.67 (13)	O2B^xv^—Sr2—O2B—Sr2^vii^	0.0
Ba1B^vii^—Ba1B—O1—Nb1	−93.29 (19)	O2B^ix^—Sr2—O2B—Sr2^vii^	57.70 (7)
Ba1B^viii^—Ba1B—O1—Nb1	170.04 (18)	O3A^iii^—Ba1—O1—Ba1^vii^	−111.7 (3)
Ba1B^i^—Ba1B—O1—Nb1	86.71 (19)	O3A^iv^—Ba1—O1—Ba1^vii^	111.7 (3)
Ba1B^i^—Ba1B—O1—Nb1^viii^	−72.6 (3)	O3A^v^—Ba1—O1—Ba1^vii^	−30.27 (11)
Ba1B^vii^—Ba1B—O1—Nb1^viii^	107.4 (3)	O3A^vi^—Ba1—O1—Ba1^vii^	30.27 (11)
Ba1B^viii^—Ba1B—O1—Nb1^viii^	10.75 (19)	O3A^iv^—Ba1—O1—Ba1B^vii^	104.8 (3)
Ba1B^vii^—Ba1B—O1—Sr1^vii^	6.69 (12)	O3A^vi^—Ba1—O1—Ba1B^xvi^	37.22 (17)
Ba1B^viii^—Ba1B—O1—Sr1^vii^	−89.99 (3)	O3A^iv^—Ba1—O1—Ba1B^xvi^	118.7 (4)
Ba1B^i^—Ba1B—O1—Sr1^vii^	−173.32 (12)	O3A^iii^—Ba1—O1—Ba1B^xvi^	−104.8 (3)
Nb1—Ba1B—O1—Ba1^vii^	99.97 (17)	O3A^v^—Ba1—O1—Ba1B^xvi^	−23.31 (16)
Nb1—Ba1B—O1—Ba1	−156.1 (16)	O3A^iii^—Ba1—O1—Ba1B^vii^	−118.7 (4)
Nb1^ii^—Ba1B—O1—Ba1	−42.7 (15)	O3A^vi^—Ba1—O1—Ba1B^vii^	23.31 (16)
Nb1^i^—Ba1B—O1—Ba1^vii^	153.76 (8)	O3A^v^—Ba1—O1—Ba1B^vii^	−37.22 (17)
Nb1^vi^—Ba1B—O1—Ba1^vii^	53.13 (12)	O3A^iv^—Ba1—O1—Ba1B^viii^	−172.1 (17)
Nb1^iv^—Ba1B—O1—Ba1	−150.0 (19)	O3A^iii^—Ba1—O1—Ba1B^viii^	−35.6 (15)
Nb1^iv^—Ba1B—O1—Ba1^vii^	106.1 (3)	O3A^vi^—Ba1—O1—Ba1B^viii^	106.4 (16)
Nb1^ii^—Ba1B—O1—Ba1^vii^	−146.66 (8)	O3A^v^—Ba1—O1—Ba1B^viii^	45.9 (16)
Nb1^vi^—Ba1B—O1—Ba1	157.1 (17)	O3A^iv^—Ba1—O1—Ba1B	35.6 (15)
Nb1^i^—Ba1B—O1—Ba1	−102.3 (16)	O3A^iii^—Ba1—O1—Ba1B	172.1 (17)
Nb1^ii^—Ba1B—O1—Ba1B^vii^	−153.35 (6)	O3A^v^—Ba1—O1—Ba1B	−106.4 (16)
Nb1^vi^—Ba1B—O1—Ba1B^viii^	143.11 (10)	O3A^vi^—Ba1—O1—Ba1B	−45.9 (16)
Nb1^iv^—Ba1B—O1—Ba1B^viii^	−163.9 (3)	O3A^iii^—Ba1—O1—Nb1	147.5 (3)
Nb1^vi^—Ba1B—O1—Ba1B^vii^	46.4 (2)	O3A^iv^—Ba1—O1—Nb1	10.9 (5)
Nb1^i^—Ba1B—O1—Ba1B^vii^	147.08 (9)	O3A^vi^—Ba1—O1—Nb1	−70.5 (2)
Nb1—Ba1B—O1—Ba1B^xvi^	107.1 (2)	O3A^v^—Ba1—O1—Nb1	−131.1 (2)
Nb1^vi^—Ba1B—O1—Ba1B^xvi^	60.25 (7)	O3A^iv^—Ba1—O1—Nb1^viii^	−147.5 (3)
Nb1^iv^—Ba1B—O1—Ba1B^vii^	99.4 (4)	O3A^iii^—Ba1—O1—Nb1^viii^	−10.9 (5)
Nb1—Ba1B—O1—Ba1B^vii^	93.29 (19)	O3A^vi^—Ba1—O1—Nb1^viii^	131.1 (2)
Nb1^i^—Ba1B—O1—Ba1B^xvi^	160.89 (17)	O3A^v^—Ba1—O1—Nb1^viii^	70.5 (2)
Nb1^ii^—Ba1B—O1—Ba1B^viii^	−56.68 (9)	O3A^iv^—Ba1—O1—Sr1^vii^	111.7 (3)
Nb1^iv^—Ba1B—O1—Ba1B^xvi^	113.2 (3)	O3A^iii^—Ba1—O1—Sr1^vii^	−111.7 (3)
Nb1^ii^—Ba1B—O1—Ba1B^xvi^	−139.54 (19)	O3A^vi^—Ba1—O1—Sr1^vii^	30.27 (11)
Nb1^i^—Ba1B—O1—Ba1B^viii^	−116.25 (7)	O3A^v^—Ba1—O1—Sr1^vii^	−30.27 (11)
Nb1—Ba1B—O1—Ba1B^viii^	−170.04 (18)	O3A^iii^—Ba1—O5A—Nb2^vii^	150.14 (12)
Nb1^ii^—Ba1B—O1—Nb1^viii^	−45.9 (2)	O3A^v^—Ba1—O5A—Nb2	−140.39 (14)
Nb1^iv^—Ba1B—O1—Nb1^viii^	−153.2 (3)	O3A^vi^—Ba1—O5A—Nb2	140.39 (14)
Nb1^iv^—Ba1B—O1—Nb1	6.1 (4)	O3A^iv^—Ba1—O5A—Nb2^vii^	−150.14 (12)
Nb1^ii^—Ba1B—O1—Nb1	113.36 (18)	O3A^iii^—Ba1—O5A—Nb2	−29.86 (12)
Nb1^i^—Ba1B—O1—Nb1^viii^	−105.5 (2)	O3A^vi^—Ba1—O5A—Nb2^vii^	−39.61 (14)
Nb1—Ba1B—O1—Nb1^viii^	−159.3 (4)	O3A^iv^—Ba1—O5A—Nb2	29.86 (12)
Nb1^vi^—Ba1B—O1—Nb1^viii^	153.9 (2)	O3A^v^—Ba1—O5A—Nb2^vii^	39.61 (14)
Nb1^i^—Ba1B—O1—Nb1	53.79 (18)	O3A—Nb1—O1—Ba1^vii^	142.7 (2)
Nb1^vi^—Ba1B—O1—Nb1	−46.8 (2)	O3A—Nb1—O1—Ba1	−122.6 (2)
Nb1^i^—Ba1B—O1—Sr1^vii^	153.76 (8)	O3A—Nb1—O1—Ba1B^viii^	−123.0 (3)
Nb1—Ba1B—O1—Sr1^vii^	99.97 (17)	O3A—Nb1—O1—Ba1B^xvi^	142.3 (3)
Nb1^ii^—Ba1B—O1—Sr1^vii^	−146.66 (8)	O3A—Nb1—O1—Ba1B^vii^	146.4 (2)
Nb1^iv^—Ba1B—O1—Sr1^vii^	106.1 (3)	O3A—Nb1—O1—Ba1B	−125.8 (3)
Nb1^vi^—Ba1B—O1—Sr1^vii^	53.13 (12)	O3A—Nb1—O1—Nb1^viii^	23.3 (5)
Nb2—Ba1—O1—Ba1^vii^	0.0	O3A—Nb1—O1—Sr1^vii^	142.7 (2)
Nb2—Ba1—O1—Ba1B^vii^	−6.96 (12)	O3A—Nb1—O2A—Ba1^vii^	162.1 (11)
Nb2—Ba1—O1—Ba1B^viii^	76.2 (16)	O3A—Nb1—O2A—Sr2	−4.6 (13)
Nb2—Ba1—O1—Ba1B	−76.2 (16)	O3A—Nb1—O2A—Sr2^vii^	−97.8 (12)
Nb2—Ba1—O1—Ba1B^xvi^	6.96 (12)	O3A—Nb1—O2B—Nb1^vi^	−166.4 (7)
Nb2—Ba1—O1—Nb1^viii^	100.79 (18)	O3A—Nb1—O2B—Sr2	−41.5 (7)
Nb2—Ba1—O1—Nb1	−100.79 (18)	O3A—Nb1—O2B—Sr2^vii^	−128.7 (6)
Nb2—Ba1—O1—Sr1^vii^	0.0	O3A—Nb1—O3B—Nb2^xviii^	−75.8 (12)
Nb2—Ba1—O5A—Nb2^vii^	180.0	O3A—Nb1—O3B—Sr1^ix^	49.7 (10)
Nb2—Ba1B—O1—Ba1^vii^	−54.0 (9)	O3A—Nb1—O3B—Sr1^xvii^	144.1 (11)
Nb2—Ba1B—O1—Ba1	49.9 (11)	O3A—Nb1—O4A—Ba1^xvii^	31.8 (2)
Nb2^vii^—Ba1B—O1—Ba1	96.6 (16)	O3A—Nb1—O4A—Nb1^vii^	−131.3 (10)
Nb2^vii^—Ba1B—O1—Ba1^vii^	−7.36 (12)	O3A—Nb1—O4A—Sr2^vii^	135.7 (2)
Nb2^vii^—Ba1B—O1—Ba1B^viii^	82.62 (12)	O3A—Nb1—O4B—Nb1^vii^	22.8 (9)
Nb2^vii^—Ba1B—O1—Ba1B^vii^	−14.0 (2)	O3A—Nb1—O4B—Sr1^vii^	−123.4 (2)
Nb2—Ba1B—O1—Ba1B^vii^	−60.7 (9)	O3A—Nb1—O4B—Sr2^vii^	123.7 (2)
Nb2—Ba1B—O1—Ba1B^xvi^	−46.9 (9)	O3A^xi^—Nb2—O5A—Ba1^xii^	43.73 (17)
Nb2^vii^—Ba1B—O1—Ba1B^xvi^	−0.24 (7)	O3A^x^—Nb2—O5A—Ba1^xii^	−43.73 (17)
Nb2—Ba1B—O1—Ba1B^viii^	35.9 (9)	O3A^iv^—Nb2—O5A—Ba1^xii^	136.27 (17)
Nb2^vii^—Ba1B—O1—Nb1	−107.3 (2)	O3A^iii^—Nb2—O5A—Ba1^xii^	−136.27 (17)
Nb2—Ba1B—O1—Nb1	−154.0 (8)	O3A^xi^—Nb2—O5A—Ba1	−136.27 (17)
Nb2^vii^—Ba1B—O1—Nb1^viii^	93.4 (2)	O3A^x^—Nb2—O5A—Ba1	136.27 (17)
Nb2—Ba1B—O1—Nb1^viii^	46.7 (10)	O3A^iii^—Nb2—O5A—Ba1	43.73 (17)
Nb2—Ba1B—O1—Sr1^vii^	−54.0 (9)	O3A^iv^—Nb2—O5A—Ba1	−43.73 (17)
Nb2^vii^—Ba1B—O1—Sr1^vii^	−7.36 (12)	O3A^iii^—Nb2—O5B—Nb2^vii^	−137.53 (17)
Sr2^vii^—Nb1—O1—Ba1	87.5 (2)	O3A^x^—Nb2—O5B—Nb2^vii^	−46.3 (2)
Sr2^vii^—Nb1—O1—Ba1^vii^	−7.2 (2)	O3A^xi^—Nb2—O5B—Nb2^vii^	46.3 (2)
Sr2^vii^—Nb1—O1—Ba1B^viii^	87.1 (3)	O3A^iv^—Nb2—O5B—Nb2^vii^	137.53 (17)
Sr2^vii^—Nb1—O1—Ba1B	84.3 (3)	O3A^xi^—Nb2—O5B—O5B^xii^	46.3 (2)
Sr2^vii^—Nb1—O1—Ba1B^xvi^	−7.6 (3)	O3A^iii^—Nb2—O5B—O5B^xii^	−137.53 (17)
Sr2^vii^—Nb1—O1—Ba1B^vii^	−3.5 (2)	O3A^x^—Nb2—O5B—O5B^xii^	−46.3 (2)
Sr2^vii^—Nb1—O1—Nb1^viii^	−126.6 (4)	O3A^iv^—Nb2—O5B—O5B^xii^	137.53 (17)
Sr2^vii^—Nb1—O1—Sr1^vii^	−7.2 (2)	O3B—Nb1—O1—Ba1^vii^	128.4 (2)
Sr2^vii^—Nb1—O2A—Ba1^vii^	−100.1 (2)	O3B—Nb1—O1—Ba1	−136.9 (2)
Sr2^vii^—Nb1—O2A—Sr2	93.24 (19)	O3B—Nb1—O1—Ba1B	−140.0 (3)
Sr2^vii^—Nb1—O2B—Nb1^vi^	−37.7 (8)	O3B—Nb1—O1—Ba1B^viii^	−137.3 (3)
Sr2^vii^—Nb1—O2B—Sr2	87.28 (17)	O3B—Nb1—O1—Ba1B^xvi^	128.0 (3)
Sr2^vii^—Nb1—O3A—Ba1^ix^	−70.9 (3)	O3B—Nb1—O1—Ba1B^vii^	132.1 (2)
Sr2^vii^—Nb1—O3A—Ba1^xvii^	16.7 (3)	O3B—Nb1—O1—Nb1^viii^	9.0 (5)
Sr2^vii^—Nb1—O3A—Nb2^xviii^	106.8 (4)	O3B—Nb1—O1—Sr1^vii^	128.4 (2)
Sr2^vii^—Nb1—O3B—Nb2^xviii^	142.8 (5)	O3B—Nb1—O2A—Ba1^vii^	−123.3 (14)
Sr2^vii^—Nb1—O3B—Sr1^xvii^	2.8 (3)	O3B—Nb1—O2A—Sr2	70.1 (15)
Sr2^vii^—Nb1—O3B—Sr1^ix^	−91.7 (2)	O3B—Nb1—O2A—Sr2^vii^	−23.1 (15)
Sr2^vii^—Nb1—O4A—Ba1^xvii^	−103.9 (2)	O3B—Nb1—O2B—Nb1^vi^	−140.1 (10)
Sr2^vii^—Nb1—O4A—Nb1^vii^	93.0 (10)	O3B—Nb1—O2B—Sr2^vii^	−102.4 (12)
Sr2^vii^—Nb1—O4B—Nb1^vii^	−100.9 (8)	O3B—Nb1—O2B—Sr2	−15.2 (13)
Sr2^vii^—Nb1—O4B—Sr1^vii^	112.9 (2)	O3B—Nb1—O3A—Ba1^xvii^	−28.6 (9)
O1^i^—Ba1—O1—Ba1^vii^	180.0	O3B—Nb1—O3A—Ba1^ix^	−116.2 (11)
O1^i^—Ba1—O1—Ba1B^vii^	173.04 (12)	O3B—Nb1—O3A—Nb2^xviii^	61.4 (9)
O1^i^—Ba1—O1—Ba1B^viii^	−103.8 (16)	O3B—Nb1—O4A—Ba1^xvii^	31.21 (17)
O1^i^—Ba1—O1—Ba1B^xvi^	−173.04 (12)	O3B—Nb1—O4A—Nb1^vii^	−131.9 (10)
O1^i^—Ba1—O1—Ba1B	103.8 (16)	O3B—Nb1—O4A—Sr2^vii^	135.1 (2)
O1^i^—Ba1—O1—Nb1	79.21 (17)	O3B—Nb1—O4B—Nb1^vii^	19.3 (8)
O1^i^—Ba1—O1—Nb1^viii^	−79.21 (17)	O3B—Nb1—O4B—Sr1^vii^	−126.83 (18)
O1^i^—Ba1—O1—Sr1^vii^	180.0	O3B—Nb1—O4B—Sr2^vii^	120.23 (18)
O1—Ba1—O5A—Nb2	180.0	O3B^iii^—Nb2—O5A—Ba1	41.63 (12)
O1^i^—Ba1—O5A—Nb2	0.0	O3B^iv^—Nb2—O5A—Ba1	−41.63 (12)
O1^i^—Ba1—O5A—Nb2^vii^	180.0	O3B^iii^—Nb2—O5A—Ba1^xii^	−138.37 (12)
O1—Ba1—O5A—Nb2^vii^	0.0	O3B^xi^—Nb2—O5A—Ba1^xii^	41.63 (12)
O1^i^—Ba1B—O1—Ba1^vii^	−173.32 (12)	O3B^x^—Nb2—O5A—Ba1^xii^	−41.63 (12)
O1^i^—Ba1B—O1—Ba1	−69.4 (15)	O3B^iv^—Nb2—O5A—Ba1^xii^	138.37 (12)
O1^i^—Ba1B—O1—Ba1B^viii^	−83.33 (13)	O3B^xi^—Nb2—O5A—Ba1	−138.37 (12)
O1^i^—Ba1B—O1—Ba1B^xvi^	−166.2 (2)	O3B^x^—Nb2—O5A—Ba1	138.37 (12)
O1^i^—Ba1B—O1—Ba1B^vii^	180.0	O3B^x^—Nb2—O5B—Nb2^vii^	−42.08 (13)
O1^i^—Ba1B—O1—Nb1^viii^	−72.6 (3)	O3B^iv^—Nb2—O5B—Nb2^vii^	137.66 (13)
O1^i^—Ba1B—O1—Nb1	86.71 (19)	O3B^iii^—Nb2—O5B—Nb2^vii^	−137.66 (13)
O1^i^—Ba1B—O1—Sr1^vii^	−173.32 (12)	O3B^xi^—Nb2—O5B—O5B^xii^	42.08 (13)
O1—Nb1—O2A—Ba1^vii^	38.6 (2)	O3B^x^—Nb2—O5B—O5B^xii^	−42.08 (13)
O1—Nb1—O2A—Sr2^vii^	138.7 (2)	O3B^iv^—Nb2—O5B—O5B^xii^	137.66 (13)
O1—Nb1—O2A—Sr2	−128.1 (2)	O3B^iii^—Nb2—O5B—O5B^xii^	−137.66 (13)
O1—Nb1—O2B—Nb1^vi^	97.4 (9)	O4A^iii^—Ba1—O1—Ba1^vii^	−78.44 (11)
O1—Nb1—O2B—Sr2^vii^	135.11 (19)	O4A^iv^—Ba1—O1—Ba1^vii^	78.44 (11)
O1—Nb1—O2B—Sr2	−137.6 (2)	O4A^iii^—Ba1—O1—Ba1B	−154.6 (16)
O1—Nb1—O3A—Ba1^ix^	139.8 (3)	O4A^iv^—Ba1—O1—Ba1B	2.3 (15)
O1—Nb1—O3A—Ba1^xvii^	−132.6 (2)	O4A^iv^—Ba1—O1—Ba1B^xvi^	85.39 (17)
O1—Nb1—O3A—Nb2^xviii^	−42.6 (5)	O4A^iii^—Ba1—O1—Ba1B^xvi^	−71.48 (15)
O1—Nb1—O3B—Nb2^xviii^	1.5 (6)	O4A^iii^—Ba1—O1—Ba1B^viii^	−2.3 (15)
O1—Nb1—O3B—Sr1^ix^	126.9 (2)	O4A^iii^—Ba1—O1—Ba1B^vii^	−85.39 (17)
O1—Nb1—O3B—Sr1^xvii^	−138.6 (2)	O4A^iv^—Ba1—O1—Ba1B^vii^	71.48 (15)
O1—Nb1—O4A—Ba1^xvii^	126.55 (16)	O4A^iv^—Ba1—O1—Ba1B^viii^	154.6 (16)
O1—Nb1—O4A—Nb1^vii^	−36.5 (10)	O4A^iv^—Ba1—O1—Nb1	−22.4 (2)
O1—Nb1—O4A—Sr2^vii^	−129.55 (16)	O4A^iii^—Ba1—O1—Nb1	−179.22 (18)
O1—Nb1—O4B—Nb1^vii^	116.1 (8)	O4A^iv^—Ba1—O1—Nb1^viii^	179.22 (18)
O1—Nb1—O4B—Sr1^vii^	−30.01 (16)	O4A^iii^—Ba1—O1—Nb1^viii^	22.4 (2)
O1—Nb1—O4B—Sr2^vii^	−142.94 (16)	O4A^iv^—Ba1—O1—Sr1^vii^	78.44 (11)
O2A^i^—Ba1—O1—Ba1^vii^	125.56 (12)	O4A^iii^—Ba1—O1—Sr1^vii^	−78.44 (11)
O2A^ii^—Ba1—O1—Ba1^vii^	−125.56 (12)	O4A^iii^—Ba1—O5A—Nb2	−87.96 (9)
O2A^ii^—Ba1—O1—Ba1B^xvi^	−118.60 (16)	O4A^iv^—Ba1—O5A—Nb2	87.96 (9)
O2A^i^—Ba1—O1—Ba1B^vii^	118.60 (16)	O4A^iii^—Ba1—O5A—Nb2^vii^	92.04 (9)
O2A^ii^—Ba1—O1—Ba1B^vii^	−132.52 (18)	O4A^iv^—Ba1—O5A—Nb2^vii^	−92.04 (9)
O2A^i^—Ba1—O1—Ba1B^xvi^	132.52 (18)	O4A—Nb1—O1—Ba1	140.7 (2)
O2A^i^—Ba1—O1—Ba1B	49.4 (16)	O4A^i^—Nb1—O1—Ba1	−49.5 (2)
O2A^ii^—Ba1—O1—Ba1B	158.3 (16)	O4A^i^—Nb1—O1—Ba1^vii^	−144.22 (19)
O2A^i^—Ba1—O1—Ba1B^viii^	−158.3 (16)	O4A—Nb1—O1—Ba1^vii^	46.0 (2)
O2A^ii^—Ba1—O1—Ba1B^viii^	−49.4 (16)	O4A—Nb1—O1—Ba1B^vii^	49.7 (2)
O2A^ii^—Ba1—O1—Nb1^viii^	−24.8 (2)	O4A^i^—Nb1—O1—Ba1B	−52.7 (3)
O2A^i^—Ba1—O1—Nb1^viii^	−133.65 (19)	O4A^i^—Nb1—O1—Ba1B^xvi^	−144.6 (2)
O2A^ii^—Ba1—O1—Nb1	133.65 (19)	O4A—Nb1—O1—Ba1B^viii^	140.3 (3)
O2A^i^—Ba1—O1—Nb1	24.8 (2)	O4A—Nb1—O1—Ba1B^xvi^	45.6 (3)
O2A^i^—Ba1—O1—Sr1^vii^	125.56 (12)	O4A^i^—Nb1—O1—Ba1B^viii^	−49.9 (3)
O2A^ii^—Ba1—O1—Sr1^vii^	−125.56 (12)	O4A—Nb1—O1—Ba1B	137.5 (3)
O2A^i^—Ba1—O5A—Nb2^vii^	−125.74 (14)	O4A^i^—Nb1—O1—Ba1B^vii^	−140.5 (2)
O2A^ii^—Ba1—O5A—Nb2^vii^	125.74 (14)	O4A—Nb1—O1—Nb1^viii^	−73.4 (5)
O2A^ii^—Ba1—O5A—Nb2	−54.26 (14)	O4A^i^—Nb1—O1—Nb1^viii^	96.4 (5)
O2A^i^—Ba1—O5A—Nb2	54.26 (14)	O4A^i^—Nb1—O1—Sr1^vii^	−144.22 (19)
O2A—Nb1—O1—Ba1	46.6 (3)	O4A—Nb1—O1—Sr1^vii^	46.0 (2)
O2A—Nb1—O1—Ba1^vii^	−48.1 (3)	O4A—Nb1—O2A—Ba1^vii^	−63.1 (2)
O2A—Nb1—O1—Ba1B^vii^	−44.4 (3)	O4A^i^—Nb1—O2A—Ba1^vii^	131.6 (2)
O2A—Nb1—O1—Ba1B^viii^	46.2 (4)	O4A^i^—Nb1—O2A—Sr2	−35.0 (2)
O2A—Nb1—O1—Ba1B	43.4 (3)	O4A^i^—Nb1—O2A—Sr2^vii^	−128.3 (2)
O2A—Nb1—O1—Ba1B^xvi^	−48.5 (3)	O4A—Nb1—O2A—Sr2^vii^	37.1 (3)
O2A—Nb1—O1—Nb1^viii^	−167.5 (5)	O4A—Nb1—O2A—Sr2	130.3 (2)
O2A—Nb1—O1—Sr1^vii^	−48.1 (3)	O4A^i^—Nb1—O2B—Nb1^vi^	−169.8 (9)
O2A^ix^—Nb1—O2A—Ba1^vii^	−143.76 (11)	O4A—Nb1—O2B—Nb1^vi^	−4.1 (10)
O2A^ix^—Nb1—O2A—Sr2	49.6 (3)	O4A^i^—Nb1—O2B—Sr2	−44.8 (2)
O2A^ix^—Nb1—O2A—Sr2^vii^	−43.6 (3)	O4A—Nb1—O2B—Sr2^vii^	33.6 (2)
O2A—Nb1—O2B—Nb1^vi^	−13.5 (7)	O4A^i^—Nb1—O2B—Sr2^vii^	−132.1 (2)
O2A^ix^—Nb1—O2B—Nb1^vi^	−84.2 (10)	O4A—Nb1—O2B—Sr2	120.9 (2)
O2A^ix^—Nb1—O2B—Sr2	40.7 (3)	O4A^i^—Nb1—O3A—Ba1^ix^	48.1 (3)
O2A^ix^—Nb1—O2B—Sr2^vii^	−46.6 (3)	O4A—Nb1—O3A—Ba1^xvii^	−30.8 (2)
O2A—Nb1—O2B—Sr2^vii^	24.2 (12)	O4A^i^—Nb1—O3A—Ba1^xvii^	135.7 (2)
O2A—Nb1—O2B—Sr2	111.4 (13)	O4A—Nb1—O3A—Ba1^ix^	−118.4 (3)
O2A^ix^—Nb1—O3A—Ba1^xvii^	50.2 (2)	O4A—Nb1—O3A—Nb2^xviii^	59.2 (5)
O2A^ix^—Nb1—O3A—Ba1^ix^	−37.4 (3)	O4A^i^—Nb1—O3A—Nb2^xviii^	−134.3 (5)
O2A—Nb1—O3A—Ba1^ix^	16.3 (13)	O4A^i^—Nb1—O3B—Nb2^xviii^	−90.8 (6)
O2A—Nb1—O3A—Ba1^xvii^	103.8 (12)	O4A—Nb1—O3B—Nb2^xviii^	102.0 (6)
O2A—Nb1—O3A—Nb2^xviii^	−166.1 (10)	O4A^i^—Nb1—O3B—Sr1^ix^	34.7 (2)
O2A^ix^—Nb1—O3A—Nb2^xviii^	140.2 (5)	O4A—Nb1—O3B—Sr1^xvii^	−38.1 (2)
O2A^ix^—Nb1—O3B—Nb2^xviii^	−176.4 (6)	O4A—Nb1—O3B—Sr1^ix^	−132.5 (3)
O2A—Nb1—O3B—Nb2^xviii^	163.2 (11)	O4A^i^—Nb1—O3B—Sr1^xvii^	129.1 (3)
O2A^ix^—Nb1—O3B—Sr1^xvii^	43.6 (3)	O4A^i^—Nb1—O4A—Ba1^xvii^	−16.9 (11)
O2A—Nb1—O3B—Sr1^ix^	−71.3 (15)	O4A^i^—Nb1—O4A—Nb1^vii^	180.003 (2)
O2A—Nb1—O3B—Sr1^xvii^	23.1 (15)	O4A^i^—Nb1—O4A—Sr2^vii^	87.0 (10)
O2A^ix^—Nb1—O3B—Sr1^ix^	−50.9 (3)	O4A—Nb1—O4B—Nb1^vii^	−13.0 (6)
O2A^ix^—Nb1—O4A—Ba1^xvii^	−56.1 (2)	O4A—Nb1—O4B—Sr1^vii^	−159.2 (7)
O2A—Nb1—O4A—Ba1^xvii^	−139.1 (2)	O4A—Nb1—O4B—Sr2^vii^	87.9 (7)
O2A^ix^—Nb1—O4A—Nb1^vii^	140.8 (10)	O4B—Nb1—O1—Ba1^vii^	30.43 (19)
O2A—Nb1—O4A—Nb1^vii^	57.9 (10)	O4B—Nb1—O1—Ba1	125.2 (2)
O2A^ix^—Nb1—O4A—Sr2^vii^	47.8 (2)	O4B^i^—Nb1—O1—Ba1^vii^	−130.67 (18)
O2A—Nb1—O4A—Sr2^vii^	−35.2 (2)	O4B^i^—Nb1—O1—Ba1	−35.9 (2)
O2A^ix^—Nb1—O4B—Nb1^vii^	−67.0 (8)	O4B^i^—Nb1—O1—Ba1B^vii^	−127.0 (2)
O2A—Nb1—O4B—Nb1^vii^	−149.9 (8)	O4B—Nb1—O1—Ba1B^viii^	124.7 (3)
O2A^ix^—Nb1—O4B—Sr1^vii^	146.8 (2)	O4B^i^—Nb1—O1—Ba1B^viii^	−36.4 (3)
O2A—Nb1—O4B—Sr1^vii^	63.9 (2)	O4B—Nb1—O1—Ba1B^vii^	34.1 (2)
O2A—Nb1—O4B—Sr2^vii^	−49.0 (2)	O4B^i^—Nb1—O1—Ba1B	−39.1 (2)
O2A^ix^—Nb1—O4B—Sr2^vii^	33.9 (2)	O4B—Nb1—O1—Ba1B^xvi^	30.1 (2)
O2A^xiv^—Sr2—O2A—Ba1^vii^	−47.7 (9)	O4B^i^—Nb1—O1—Ba1B^xvi^	−131.0 (2)
O2A^i^—Sr2—O2A—Ba1^vii^	8.5 (8)	O4B—Nb1—O1—Ba1B	122.0 (3)
O2A^iv^—Sr2—O2A—Ba1^vii^	64.6 (9)	O4B^i^—Nb1—O1—Nb1^viii^	109.9 (5)
O2A^i^—Sr2—O2A—Nb1^vi^	−87.2 (2)	O4B—Nb1—O1—Nb1^viii^	−89.0 (5)
O2A^i^—Sr2—O2A—Nb1	85.9 (2)	O4B^i^—Nb1—O1—Sr1^vii^	−130.67 (18)
O2A^iv^—Sr2—O2A—Nb1	142.0 (2)	O4B—Nb1—O1—Sr1^vii^	30.43 (19)
O2A^iv^—Sr2—O2A—Nb1^vi^	−31.0 (4)	O4B^i^—Nb1—O2A—Ba1^vii^	120.0 (2)
O2A^xiv^—Sr2—O2A—Nb1	29.7 (4)	O4B—Nb1—O2A—Ba1^vii^	−49.64 (19)
O2A^xiv^—Sr2—O2A—Nb1^vi^	−143.36 (19)	O4B^i^—Nb1—O2A—Sr2	−46.7 (2)
O2A^iv^—Sr2—O2A—Sr2^vii^	−123.84 (16)	O4B^i^—Nb1—O2A—Sr2^vii^	−139.9 (2)
O2A^i^—Sr2—O2A—Sr2^vii^	180.0	O4B—Nb1—O2A—Sr2^vii^	50.5 (2)
O2A^xiv^—Sr2—O2A—Sr2^vii^	123.84 (16)	O4B—Nb1—O2A—Sr2	143.7 (3)
O2A^xiv^—Sr2—O2B—Nb1	46.5 (3)	O4B—Nb1—O2B—Nb1^vi^	8.5 (9)
O2A^iv^—Sr2—O2B—Nb1^vi^	−47.3 (3)	O4B^i^—Nb1—O2B—Nb1^vi^	179.3 (10)
O2A^i^—Sr2—O2B—Nb1^vi^	−99.4 (2)	O4B^i^—Nb1—O2B—Sr2^vii^	−143.1 (2)
O2A^xiv^—Sr2—O2B—Nb1^vi^	−151.60 (16)	O4B—Nb1—O2B—Sr2^vii^	46.15 (19)
O2A^iv^—Sr2—O2B—Nb1	150.83 (17)	O4B^i^—Nb1—O2B—Sr2	−55.80 (19)
O2A^i^—Sr2—O2B—Nb1	98.7 (2)	O4B—Nb1—O2B—Sr2	133.4 (2)
O2A^i^—Sr2—O2B—Sr2^vii^	−179.98 (17)	O4B—Nb1—O3A—Ba1^ix^	−129.9 (3)
O2A^xiv^—Sr2—O2B—Sr2^vii^	127.86 (17)	O4B—Nb1—O3A—Ba1^xvii^	−42.3 (3)
O2A^iv^—Sr2—O2B—Sr2^vii^	−127.84 (19)	O4B^i^—Nb1—O3A—Ba1^xvii^	145.6 (2)
O2B^ix^—Nb1—O1—Ba1^vii^	−143.5 (7)	O4B^i^—Nb1—O3A—Ba1^ix^	58.1 (3)
O2B—Nb1—O1—Ba1^vii^	−60.7 (2)	O4B—Nb1—O3A—Nb2^xviii^	47.8 (5)
O2B^ix^—Nb1—O1—Ba1	−48.7 (8)	O4B^i^—Nb1—O3A—Nb2^xviii^	−124.3 (5)
O2B—Nb1—O1—Ba1	34.0 (2)	O4B—Nb1—O3B—Nb2^xviii^	91.4 (6)
O2B—Nb1—O1—Ba1B^xvi^	−61.1 (3)	O4B^i^—Nb1—O3B—Nb2^xviii^	−81.7 (6)
O2B^ix^—Nb1—O1—Ba1B^vii^	−139.8 (8)	O4B—Nb1—O3B—Sr1^ix^	−143.1 (2)
O2B—Nb1—O1—Ba1B^vii^	−57.0 (2)	O4B^i^—Nb1—O3B—Sr1^xvii^	138.3 (2)
O2B^ix^—Nb1—O1—Ba1B^xvi^	−143.8 (8)	O4B—Nb1—O3B—Sr1^xvii^	−48.7 (2)
O2B—Nb1—O1—Ba1B^viii^	33.6 (3)	O4B^i^—Nb1—O3B—Sr1^ix^	43.8 (2)
O2B^ix^—Nb1—O1—Ba1B^viii^	−49.2 (8)	O4B—Nb1—O4A—Ba1^xvii^	178.8 (7)
O2B—Nb1—O1—Ba1B	30.8 (3)	O4B—Nb1—O4A—Nb1^vii^	15.7 (7)
O2B^ix^—Nb1—O1—Ba1B	−51.9 (8)	O4B—Nb1—O4A—Sr2^vii^	−77.3 (6)
O2B^ix^—Nb1—O1—Nb1^viii^	97.1 (9)	O4B^i^—Nb1—O4B—Nb1^vii^	180.003 (2)
O2B—Nb1—O1—Nb1^viii^	179.9 (5)	O4B^i^—Nb1—O4B—Sr1^vii^	33.9 (9)
O2B—Nb1—O1—Sr1^vii^	−60.7 (2)	O4B^i^—Nb1—O4B—Sr2^vii^	−79.1 (8)
O2B^ix^—Nb1—O1—Sr1^vii^	−143.5 (7)	O4B^xiii^—Sr2—O2A—Ba1^vii^	142.2 (7)
O2B—Nb1—O2A—Ba1^vii^	107.9 (13)	O4B^xiv^—Sr2—O2A—Ba1^vii^	−109.5 (7)
O2B^ix^—Nb1—O2A—Ba1^vii^	−156.19 (15)	O4B^iv^—Sr2—O2A—Ba1^vii^	68.2 (7)
O2B—Nb1—O2A—Sr2^vii^	−152.0 (14)	O4B^i^—Sr2—O2A—Ba1^vii^	−35.7 (7)
O2B^ix^—Nb1—O2A—Sr2^vii^	−56.1 (2)	O4B^iv^—Sr2—O2A—Nb1^vi^	−27.5 (2)
O2B—Nb1—O2A—Sr2	−58.7 (12)	O4B^i^—Sr2—O2A—Nb1	41.66 (19)
O2B^ix^—Nb1—O2A—Sr2	37.2 (2)	O4B^iv^—Sr2—O2A—Nb1	145.5 (3)
O2B^ix^—Nb1—O2B—Nb1^vi^	−97.3 (9)	O4B^xiv^—Sr2—O2A—Nb1	−32.1 (3)
O2B^ix^—Nb1—O2B—Sr2	27.7 (3)	O4B^xiii^—Sr2—O2A—Nb1	−140.4 (2)
O2B^ix^—Nb1—O2B—Sr2^vii^	−59.6 (2)	O4B^i^—Sr2—O2A—Nb1^vi^	−131.4 (3)
O2B—Nb1—O3A—Ba1^xvii^	132.2 (6)	O4B^xiii^—Sr2—O2A—Nb1^vi^	46.5 (3)
O2B^ix^—Nb1—O3A—Ba1^ix^	−25.9 (3)	O4B^xiv^—Sr2—O2A—Nb1^vi^	154.87 (19)
O2B—Nb1—O3A—Ba1^ix^	44.6 (7)	O4B^xiii^—Sr2—O2A—Sr2^vii^	−46.3 (2)
O2B^ix^—Nb1—O3A—Ba1^xvii^	61.7 (2)	O4B^xiv^—Sr2—O2A—Sr2^vii^	62.06 (18)
O2B—Nb1—O3A—Nb2^xviii^	−137.8 (5)	O4B^i^—Sr2—O2A—Sr2^vii^	135.8 (2)
O2B^ix^—Nb1—O3A—Nb2^xviii^	151.8 (5)	O4B^iv^—Sr2—O2A—Sr2^vii^	−120.32 (18)
O2B^ix^—Nb1—O3B—Nb2^xviii^	−163.6 (6)	O4B^iv^—Sr2—O2B—Nb1	161.9 (3)
O2B—Nb1—O3B—Nb2^xviii^	−120.3 (11)	O4B^i^—Sr2—O2B—Nb1	50.22 (18)
O2B—Nb1—O3B—Sr1^xvii^	99.6 (12)	O4B^iv^—Sr2—O2B—Nb1^vi^	−36.26 (17)
O2B^ix^—Nb1—O3B—Sr1^xvii^	56.3 (2)	O4B^xiv^—Sr2—O2B—Nb1^vi^	146.70 (17)
O2B^ix^—Nb1—O3B—Sr1^ix^	−38.1 (2)	O4B^xiii^—Sr2—O2B—Nb1^vi^	29.3 (3)
O2B—Nb1—O3B—Sr1^ix^	5.1 (13)	O4B^i^—Sr2—O2B—Nb1^vi^	−147.9 (3)
O2B—Nb1—O4A—Ba1^xvii^	−141.2 (2)	O4B^xiii^—Sr2—O2B—Nb1	−132.61 (19)
O2B^ix^—Nb1—O4A—Ba1^xvii^	−51.0 (2)	O4B^xiv^—Sr2—O2B—Nb1	−15.2 (3)
O2B—Nb1—O4A—Nb1^vii^	55.7 (10)	O4B^xiii^—Sr2—O2B—Sr2^vii^	−51.3 (2)
O2B^ix^—Nb1—O4A—Nb1^vii^	145.9 (10)	O4B^i^—Sr2—O2B—Sr2^vii^	131.6 (2)
O2B—Nb1—O4A—Sr2^vii^	−37.3 (3)	O4B^xiv^—Sr2—O2B—Sr2^vii^	66.16 (17)
O2B^ix^—Nb1—O4A—Sr2^vii^	52.9 (2)	O4B^iv^—Sr2—O2B—Sr2^vii^	−116.80 (17)
O2B—Nb1—O4B—Nb1^vii^	−155.0 (8)	O5A—Ba1—O1—Ba1^vii^	0.0
O2B^ix^—Nb1—O4B—Nb1^vii^	−65.5 (8)	O5A—Ba1—O1—Ba1B^viii^	76.2 (16)
O2B^ix^—Nb1—O4B—Sr1^vii^	148.4 (2)	O5A—Ba1—O1—Ba1B^xvi^	6.96 (12)
O2B—Nb1—O4B—Sr1^vii^	58.8 (2)	O5A—Ba1—O1—Ba1B	−76.2 (16)
O2B^ix^—Nb1—O4B—Sr2^vii^	35.4 (2)	O5A—Ba1—O1—Ba1B^vii^	−6.96 (12)
O2B—Nb1—O4B—Sr2^vii^	−54.1 (2)	O5A—Ba1—O1—Nb1	−100.79 (18)
O2B^xv^—Sr2—O2A—Ba1^vii^	−171.5 (7)	O5A—Ba1—O1—Nb1^viii^	100.79 (17)
O2B^ix^—Sr2—O2A—Ba1^vii^	−110.1 (7)	O5A—Ba1—O1—Sr1^vii^	0.0
O2B^vi^—Sr2—O2A—Ba1^vii^	127.1 (8)	O5A^i^—Nb2—O5B—Nb2^vii^	180.000 (1)
O2B—Sr2—O2A—Ba1^vii^	8.3 (7)	O5A—Nb2—O5B—Nb2^vii^	0.0
O2B—Sr2—O2A—Nb1^vi^	−87.3 (12)	O5A—Nb2—O5B—O5B^xii^	0.000 (3)
O2B^vi^—Sr2—O2A—Nb1^vi^	31.40 (18)	O5A^i^—Nb2—O5B—O5B^xii^	180.000 (4)
O2B^xv^—Sr2—O2A—Nb1^vi^	92.8 (2)	O5B—Nb2—O5A—Ba1	0.0
O2B^ix^—Sr2—O2A—Nb1^vi^	154.2 (3)	O5B^xii^—Nb2—O5A—Ba1	180.000 (2)
O2B^xv^—Sr2—O2A—Nb1	−94.2 (2)	O5B^xii^—Nb2—O5A—Ba1^xii^	0.000 (2)
O2B^ix^—Sr2—O2A—Nb1	−32.76 (19)	O5B—Nb2—O5A—Ba1^xii^	180.0
O2B—Sr2—O2A—Nb1	85.7 (12)	O5B^xii^—Nb2—O5B—Nb2^vii^	0.0

Symmetry codes: (i) *x*, *y*, *z*+1; (ii) *y*+1/2, *x*−1/2, *z*+1; (iii) *x*+1/2, −*y*+1/2, *z*+1; (iv) −*y*+1, *x*, *z*+1; (v) *x*+1/2, −*y*+1/2, *z*; (vi) −*y*+1, *x*, *z*; (vii) *x*, *y*, *z*−1; (viii) *y*+1/2, *x*−1/2, *z*; (ix) *y*, −*x*+1, *z*; (x) *y*+1, −*x*+1, *z*+1; (xi) −*x*+3/2, *y*+1/2, *z*+1; (xii) −*x*+2, −*y*+1, *z*; (xiii) −*x*+1, −*y*+1, *z*+1; (xiv) *y*, −*x*+1, *z*+1; (xv) −*x*+1, −*y*+1, *z*; (xvi) *y*+1/2, *x*−1/2, *z*−1; (xvii) *y*, −*x*+1, *z*−1; (xviii) −*y*+1, *x*−1, *z*−1.
